# Renal mitochondria response to sepsis: a sequential biopsy evaluation of experimental porcine model

**DOI:** 10.1186/s40635-025-00732-0

**Published:** 2025-02-22

**Authors:** Jiri Müller, Jiri Chvojka, Lenka Ledvinova, Jan Benes, Zdenek Tuma, Martina Grundmanova, Jan Jedlicka, Jitka Kuncova, Martin Matejovic

**Affiliations:** 1 1st Department of Internal Medicine, Faculty of Medicine in Pilsen, Teaching Hospital, Charles University, Prague Alej Svobody 80, 32300 Pilsen, Czech Republic; 2 Laboratory of Experimental Intensive Care Medicine, Biomedical Center, Faculty of Medicine in Pilsen, Charles University, Prague, Czech Republic; 3 Department of Anesthesiology, Resuscitation and Intensive Care, Faculty of Medicine in Pilsen, Charles University, Prague, Czech Republic; 4 Proteomic Laboratory, Biomedical Center, Faculty of Medicine in Pilsen, Charles University, Prague, Czech Republic; 5 Department of Physiology, Faculty of Medicine in Pilsen, Charles University, Prague, Czech Republic; 6 Mitochondrial Laboratory, Biomedical Center, Faculty of Medicine in Pilsen, Charles University, Prague, Czech Republic

**Keywords:** Sepsis, Septic shock, Acute kidney injury, Mitochondrial dysfunction, Renal biopsy

## Abstract

**Background:**

The pathophysiology of sepsis-induced acute kidney injury remains elusive. Although mitochondrial dysfunction is often perceived as the main culprit, data from preclinical models yielded conflicting results so far. The aim of this study was to assess the immune-metabolic background of sepsis-associated renal dysfunction using sequential biopsy approach with mitochondria function evaluation in a large clinically relevant porcine models mimicking two different paces and severity of sepsis and couple this approach with traditional parameters of renal physiology.

**Methods:**

In this randomized, open-label study, 15 anaesthetized, mechanically ventilated and instrumented (renal artery flow probe and renal vein catheter) pigs were randomized in two disease severity groups—low severity (LS) sepsis (0.5 g/kg of autologous faeces intraperitoneally) and high severity (HS) sepsis (1 g/kg of autologous faeces intraperitoneally). Sequential cortical biopsies of the left kidney were performed and a pyramid-shaped kidney specimen with cortex, medulla and renal papilla was resected and processed at the end of the experiment. Oxygraphic data and western blot analysis of proteins involved in mitochondrial biogenesis and degradation were obtained.

**Results:**

In contrast to increased mitochondrial activity observed in LS sepsis, a significant decrease in the oxidative phosphorylation capacity together with an increase in the respiratory system uncoupling was observed during the first 24 h after sepsis induction in the HS group. Those changes preceded alterations of renal haemodynamics. Furthermore, serum creatinine rose significantly during the first 24 h, indicating that renal dysfunction is not primarily driven by haemodynamic changes. Compared to cortex, renal medulla had significantly lower oxidative phosphorylation capacity and electron-transport system activity. PGC-1-alfa, a marker of mitochondrial biogenesis, was significantly decreased in HS group.

**Conclusions:**

In this experimental model, unique sequential tissue data show that the nature and dynamics of renal mitochondrial responses to sepsis are profoundly determined by the severity of infectious challenge and resulting magnitude of inflammatory insult. High disease severity is associated with early and stepwise progression of mitochondria dysfunction and acute kidney injury, both occurring independently from later renal macro-haemodynamic alterations. Our data may help explain the conflicting results of preclinical studies and suggest that sepsis encompasses a very broad spectrum of sepsis-induced acute kidney injury endotypes.

**Supplementary Information:**

The online version contains supplementary material available at 10.1186/s40635-025-00732-0.

## Introduction

Sepsis is dysregulated response of host immune system to infection [1] and represents the main cause of acute kidney injury in critical illness, accounting for 25–75% of the cases of acute renal failure [2, 3]. Moreover, the incidence of septic AKI rises proportionally with the severity of sepsis [4]. Development of renal dysfunction during sepsis and septic shock is associated with poor outcome and represents an independent predictor of both short-term and long-term mortality and morbidity [5–7]. Although the connection between sepsis and renal dysfunction is well known, precise underlying mechanisms remain largely elusive. The cardiovascular thesis of microvascular dysfunction, blood shunting and hypoperfusion with tissue hypoxia as a sole causative factor remains controversial, since numerous studies have shown high tissue oxygen tensions within failing septic organs [8, 9] and only minimal cell injury in organ biopsies, despite the clear evidence of inflammation [10]. Similarly, septic acute kidney injury is associated with only minimal changes of histologic morphology in renal biopsies [11, 12] and reduced renal blood flow is not necessary for renal dysfunction development [13]. Therefore, yet another reason for sepsis-driven renal injury must exist. Growing body of evidence has associated organ dysfunction with a complex cellular immune-metabolic reprogramming and mitochondria dysfunction [14, 15]. However, current research regarding pathophysiology of those subcellular processes yielded conflicting results so far, reflecting a complex septic AKI endotyping dependent on multiple variables such as a degree of proinflammatory insult, type of causative pathogen, genetic background rendering susceptibility to kidney injury and dynamic nature of septic AKI. The main goal of this study was to assess renal mitochondrial function using sequential biopsy approach in two different models of sepsis severity and compare these results with traditional parameters of renal physiology. We aimed to test a hypothesis that renal dysfunction, at least in some endotypes of septic AKI, is caused by functional adaptation of cell metabolism and mitochondria in an immune-metabolic manner and to investigate whether these changes precede haemodynamic deterioration in septic shock.

## Methods

The experiment was conducted in the laboratory of experimental intensive care at the Biomedical Center facility, Faculty of Medicine in Pilsen, Charles University, Czech Republic. All experiments were handled in accordance with the European Directive for the Protection of Animals Used for Experimental Purposes and approved by the University Animal Care Committee and by the Ministry of Education, Youth and Sports of the Czech Republic. The ARRIVE Guidelines were followed [16]. The animals were obtained from a local breeder as established in our facility. They were quarantined for two weeks with daily check-ups. During this time, they were fed with dry granules and had unlimited access to water. The animals were fasted 12 h prior to instrumentation. A total of fifteen domestic pigs (*Sus scrofa f. domestica*) of both sexes with median body weight of 41[36–46] kg and an age of 2–4 months were enrolled to the study. Animals were allocated to groups by simple randomization on a 1:1 basis. As there was no treatment intervention and identical supportive care with antibiotics was provided, no specific randomization method was used. However, animals were selected by a veterinarian who was blinded to group allocation. Due to the interventional nature of the study, the investigators performing the instrumentation and subsequent experiment were not blinded. All laboratory measurements and statistical analyses were performed independently.

### Anaesthesia and instrumentation

Anaesthesia and instrumentation were similar to those previously described [17, 18]. All animals were anaesthetized with xylazine (1 mg/kg) and tiletamine–zolazepam (2.2 mg/kg). A 1–2 mg/kg bolus dose of 2% propofol was administered after peripheral venous line insertion. Animals were then intubated and mechanically ventilated. For ventilation volume-control mode was used with tidal volumes of 8‒10 ml/kg, PEEP of 8 cmH_2_O, FiO_2_ of 0.3 and respiratory rate adjusted to maintain normocapnia. During surgery anaesthesia was maintained by continuous administration of 2% propofol (4–6 mg/kg/h) and fentanyl (8‒10 µg/kg/h). Muscle relaxation was maintained by bolus (0.6 mg/kg) and then continuous administration of rocuronium (0.2–0.4 mg/kg/h). Drug dosing was halved after surgery with a discontinuation of rocuronium. Continuous infusion of Ringerfundin solution (B. Braun Melsungen AG, Germany) was used as fluid replacement at a dose of 10 mL/kg/h during surgery and 5 mL/kg/h afterwards. Normoglycemia was maintained by continuous infusion of 10% glucose. Before surgical procedure, femoral artery catheter with thermistor, Swan–Ganz catheter and central venous line were inserted for haemodynamic measurement via PiCCO device. Midline laparotomy was then performed, and a pre-calibrated ultrasound flow probe (Transonic Systems, Ithaca, NY) was placed around the left renal artery. A catheter was inserted into the left renal vein for renal venous pressure measurements and blood sampling. Two drains were inserted for peritoneal drainage and autologous faecal inoculation. A curved linear incision was made caudal to the last rib and left kidney was manually exposed without entering the abdominal cavity. This allowed for visually controlled repeated kidney biopsies. A recovery period of six hours was provided before the baseline measurement.

### Experimental protocol

**Fig. 1:**
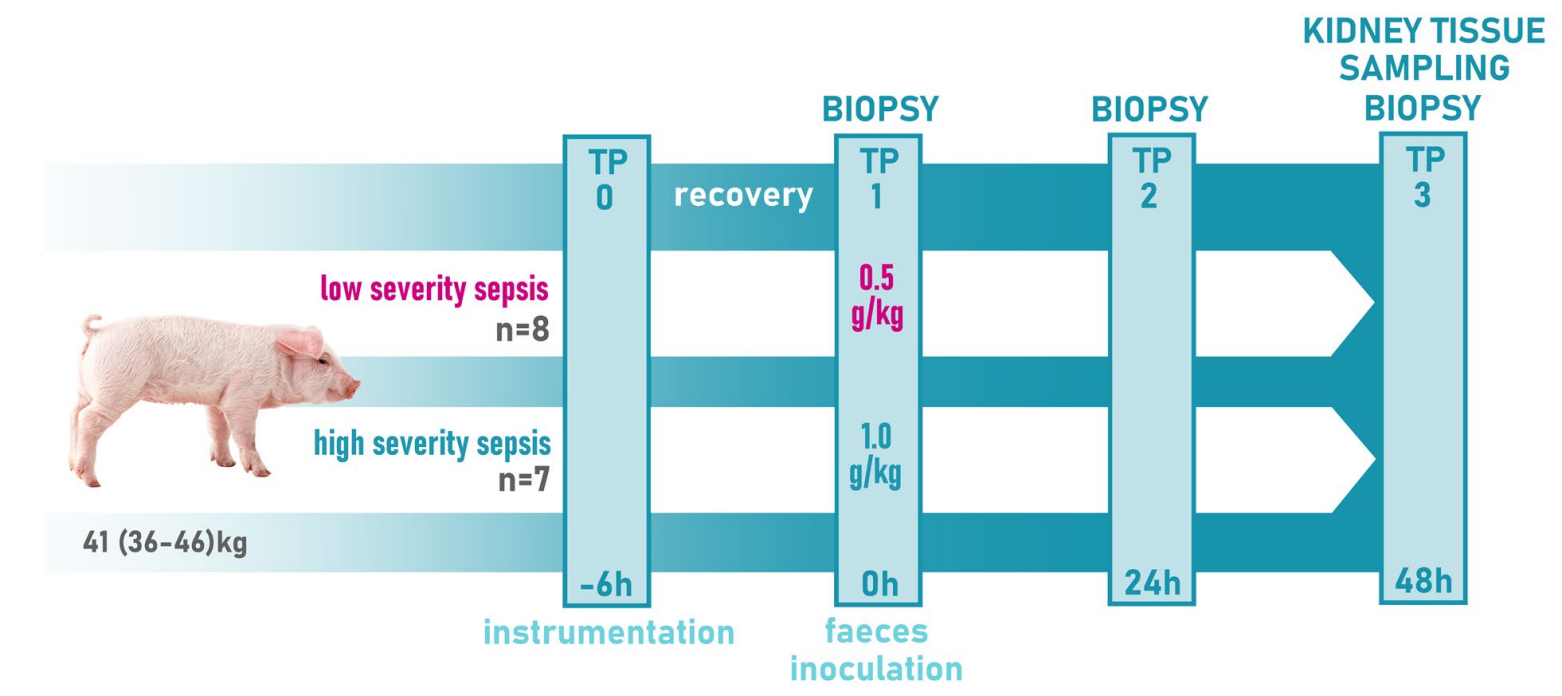
Study protocol flow-chart. TP—time point

In order to investigate two distinct dynamics of sepsis progression, the study consisted of two arms determined by the intensity of infectious load: (1) low severity sepsis (LS-sepsis) induced by an intraperitoneal inoculation with 0.5 g/kg of autologous faeces cultivated 4 h in 200 mL of saline at 38 °C (n = 8), and (2) high severity sepsis (HS-sepsis) induced by an intraperitoneal inoculation with 1 g/kg of autologous faeces (n = 7). Sepsis was defined as a systemic inflammation accompanied with organ dysfunction (assessed by the Sequential Organ Failure Assessment score with exclusion of the Glasgow Coma Scale-based neurologic component) and typical haemodynamical changes associated with hyperdynamic circulation. Repeated crystalloid boluses (Ringerfundin B. Braun Melsungen AG, Germany) were used to optimize volume status based on the invasive haemodynamic measurements. Norepinephrine was used as a vasopressor whenever mean arterial pressure fell below 65 mmHg despite adequate fluid therapy. Each animal was treated with intravenous antibiotics (piperacillin–tazobactam 2.25 g every 8 h). At the end of the experiment, animals were euthanized by anaesthetic overdose and excision of the heart (Fig. 1).

### Measurements, monitoring, and sampling

At each time-point (baseline, 24 and 48 h after sepsis induction) datasets including measurements and calculations of systemic and regional haemodynamics, fluids and vasopressor requirement were collected. Arterial and mixed venous blood samples were analysed. Complete blood count and other biochemical parameters were obtained. (See *Additional material 1*) Cortical biopsies of the left kidney were sequentially performed at each time-point. At the end of the experiment (time-point 3; 48 h after the induction), a pyramid-shaped kidney specimen showing cortex, medulla and renal papilla was resected and processed.

### Evaluation of mitochondrial respiration

Mitochondrial respiration was assessed in mechanically permeabilized biopsies using high-resolution respirometry (Oxygraph O2k, Oroboros, Innsbruck, Austria). Swine kidney cortex and medulla tissue samples were mechanically permeabilized and washed in mitochondrial respiration medium. Samples were placed into pre-calibrated oxygraph chambers and oxygen consumptions were analysed and adjusted to the ROX (residual oxygen consumption) state—oxygen consumption after complete inhibition of the electron transfer system. To evaluate functional state of mitochondrial respiration, a protocol with sequential titration of substrates and inhibitors of electron-transferring complexes was performed. The end-point kidney samples were frozen and then the enzymatic activity of citrate synthase was measured simultaneously in all samples at the end of the study. Capacity of the mitochondrial respiratory system was analysed in phosphorylating coupled states for complex I and I + II (PI, PI + II); uncoupled oxygen consumption was determined for complexes I and II (EI + II), II (EII), and IV (CIV) after appropriate addition of substrates, inhibitors and uncoupler. Several calculated parameters and ratios were determined to further evaluate efficiency and quality of the mitochondrial electron-transporting system. See *Additional material 2* for details.

### Western blot analysis

Western blot analysis was analogous to our previous studies [21]. Proteins from kidney tissue homogenates were separated by sodium dodecyl-sulphate polyacrylamide gel electrophoresis and then transferred onto a PVDF membrane (Sigma, Steinheim, Germany). The membranes were blocked in TBST (0.15 mol/L NaCl, 20 mmol/L Tris–HCl, pH 7.5 with 0.1% Tween-20 and 5% non-fat dry milk) and incubated overnight. Primary antibodies against PGC-1-alpha (Abcam, ab 191,838) and against LC3A/B (microtubule-associated protein light chain 3; Cell Signalling technologies, ab 4108S) were used for detection of corresponding proteins. The membranes were subsequently incubated with secondary antibody (Abcam ab6721). Detection was performed with Opti-4CN kit (Bio-Rad Laboratories, California, USA). Intensities of bands acquired by Western blotting were normalized to total protein.

### Statistical analysis

All values shown are median with interquartile range if not indicated otherwise. Statistical analysis was carried out using STATISTICA 12 (TIBCO Software Inc., California, USA). After exclusion of normality (Shapiro–Wilk’s test), time-dependent changes within groups were tested by Friedman ANOVA with appropriate post hoc test and/or Mann–Whitney signed-rank test. Differences between groups were assessed using the Mann–Whitney rank-sum test. A p value < 0.05 was considered statistically significant.

## Results

### Characteristics of sepsis models

**Fig. 2:**
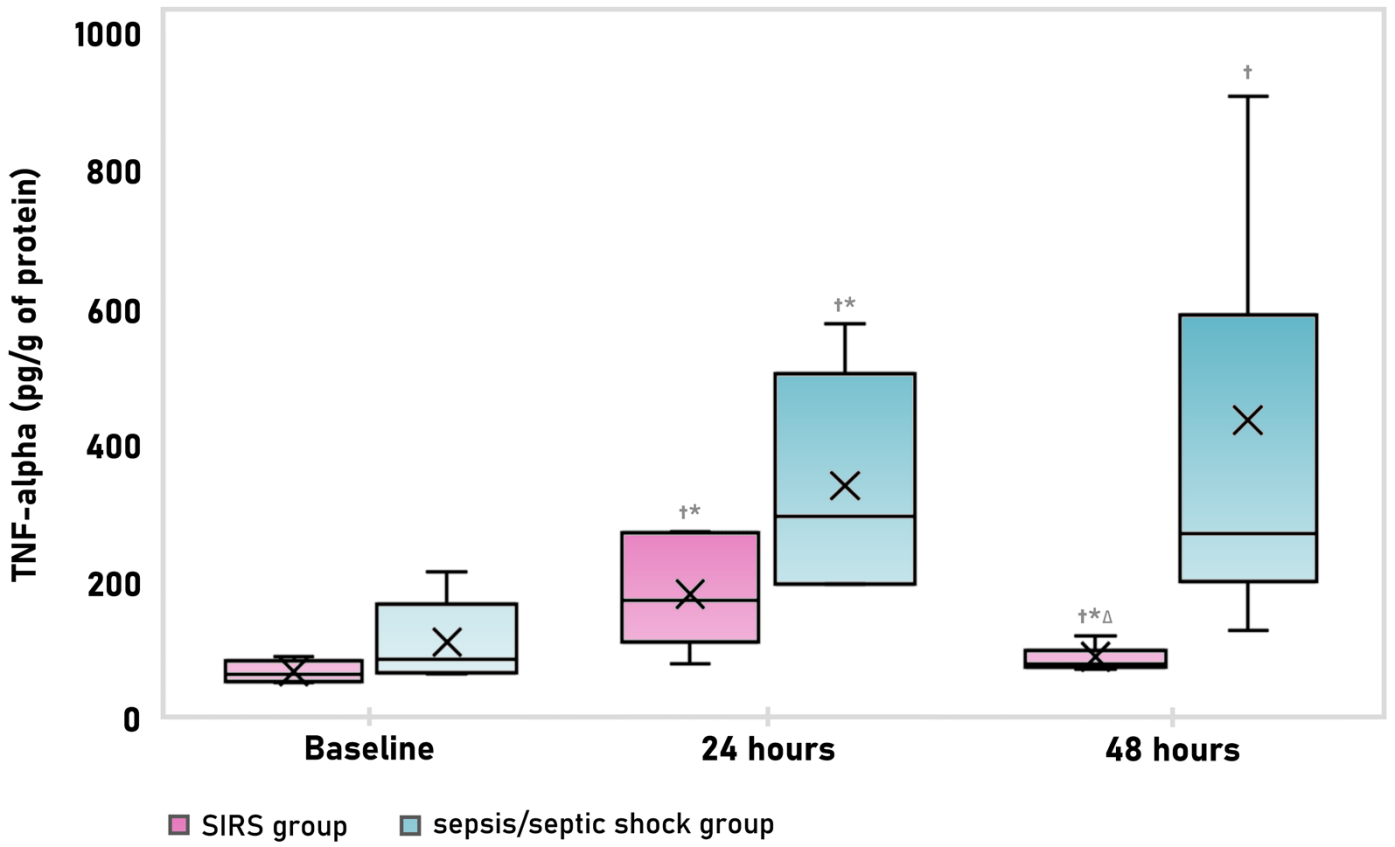
TNF-alfa dynamics in both low severity (pink) and high severity sepsis (blue) group. * Significant change from the preceding time-point, ^∆^significant difference from the baseline, ^†^ significant difference between groups

Haemodynamics and inflammatory parameters are summarized in *Additional material *3 (Table A1 and Table A2). All subjects developed hyperdynamic circulation with increased cardiac output and decreased systemic vascular resistance. Those changes were more prominent in the high severity sepsis group as expected. Progressive and persistent increase in proinflammatory cytokines (IL-6 and TNF-alfa) was more pronounced in high severity sepsis compared to a rather transient pattern observed in low severity group (Fig. 2*, and Additional material* Table A2).

**Fig. 3:**
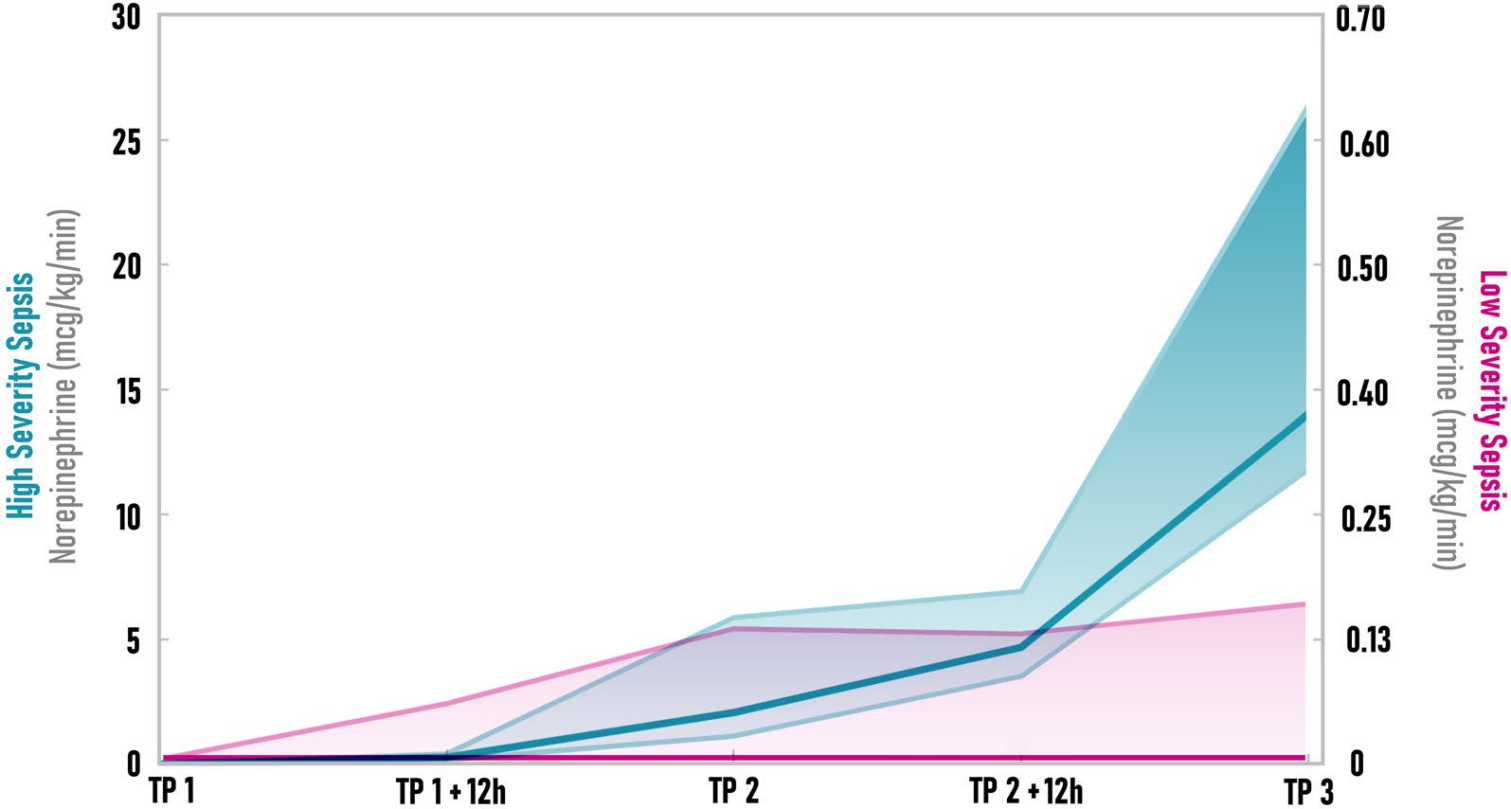
Norepinephrine infusion rate progression. Curve depicts the median value for the group in given time-points. Shaded area corresponds to the 25–75 quartile values. Low severity (LS) sepsis in pink and high severity (HS) sepsis in blue. TP—time point

Modified SOFA score parameters for both low and high severity sepsis groups are summarized in Table 1. Norepinephrine dosage was significantly higher in the high severity sepsis group compared to the low severity group (Fig. 3).

**Table 1:** Modified SOFA score parameters

System parameter	Group	Baseline	24 h	48 h
Respiration (PaO_2_/FiO_2_)	LS sepsis	449 (411–489)	249 (222–305)^*∆†^	252 (230–297)^∆^
	HS sepsis	461 (459–483)	103 (100–183)^*∆^	183 (148–224)
Platelets (× 10^9^/L)	LS sepsis	239 (223–247)	149 (121–182)^*∆^	117 (89–144)^∆^
	HS sepsis	286 (184–395)	196 (141–235)^*∆^	89 (71–137)
Bilirubin (µmol/L)	LS sepsis	3 (3–3)	3 (3–3)	3 (3–3)
	HS sepsis	3 (3–3)	3 (3–3)	3 (3–3)
Mean arterial pressure (mmHg)	LS sepsis	78 (74–97)	78 (74–79)	78 (77–79)^†^
	HS sepsis	80 (67–85)	71 (66–73)	52 (41–54)^†^
Creatinine (umol/L)	LS sepsis	83 (73–97)	79 (73–99)^†^	77 (67–83)^†^
	HS sepsis	102 (93–117)	122 (95–161)^*^	180 (164–197)^†^
Diuresis (mL/kg/h)	LS sepsis	1 (0.8–1.1)	2.1 (1.3–2.7)	3.6 (3.2–4.1)^*∆†^
	HS sepsis	1.4 (1.2–2.3)	1.4 (0.9–1.9)	2.5 (1.6–2.8)^†^

All values shown are median with interquartile range

LS—low severity. HS—high severity

*Significant change from the preceding time-point

^∆^significant difference from the baseline

^†^significant difference between groups

### Renal haemodynamics and response to systemic infection and sepsis

**Fig. 4:**
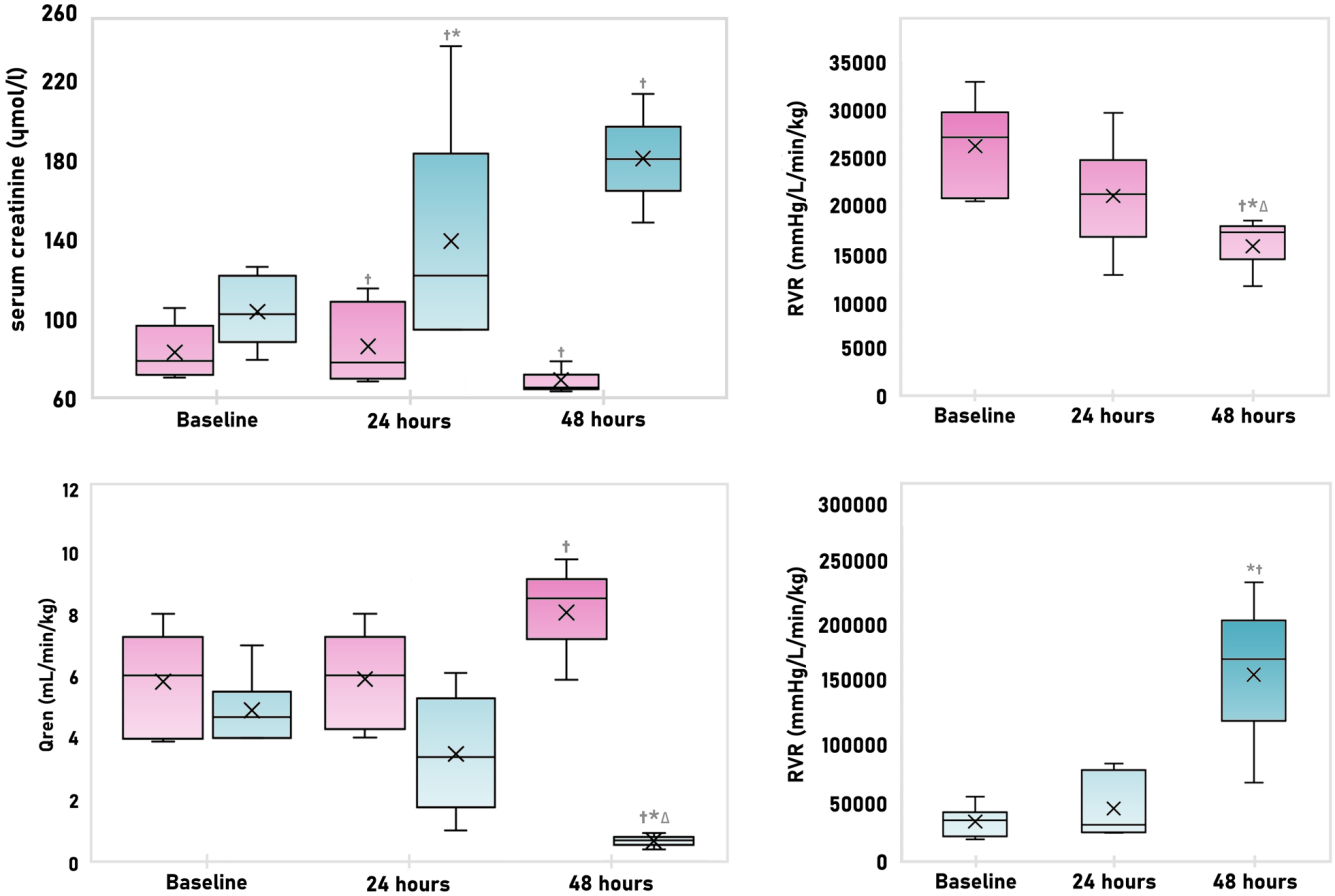
Renal haemodynamics and serum creatinine in response to LS (low severity) sepsis (pink) and HS (high severity) sepsis (blue). Q_ren_—renal blood flow; RVR—renal vascular resistance; * significant change from the preceding time-point, ^∆^significant difference from the baseline, ^†^ significant difference between groups

Renal haemodynamics and acute kidney injury parameters are briefly summarized in *Additional *material (Table A3). Renal blood flow reduction associated with a significant increase in renal vascular resistance was observed in HS sepsis animals with the biggest change between 24 to 48 h time-point from the sepsis induction. In the LS sepsis group renal vascular resistance decreased, but no statistically significant decrease in renal perfusion occurred. Animals with HS sepsis therefore developed notable alterations in renal haemodynamics in comparison to animals from the LS sepsis group. Unlike in LS sepsis, in HS septic animals serum creatinine increased. Changes in serum creatinine preceded those in renal perfusion and vascular resistance suggesting other non-haemodynamic factors in the acute kidney injury development. No animals in LS sepsis group met the criteria for acute kidney injury. Selected variables are depicted in Fig. 4.

### Renal high-resolution respirometry in sequential biopsies

Time-dependent changes of renal mitochondrial respiration are summarized in Table 2*.*

**Table 2:** High-resolution respirometry in sequential kidney tissue biopsies

Respirometry parameters (pmol O_2_/s/mg)	Group	Baseline	24 h	48 h
L_I_	LS sepsis	3 (1.9–3.6)	3.3 (1.5–5.9)	4.9 (4.5–6.5)
	HS sepsis	1.5 (1.1–2.3)	1.6 (1.5–2.1)	3.5 (3.4–5.5)
P_I_	LS sepsis	33.6 (28.3–37.9)	38.2 (35.6–42.6)^†^	35.8 (29.9–43.4)
	HS sepsis	30.0 (28–37.2)	20.4 (18.9–25.5)^*^	16.7 (16.2–24.9)
P_I+II_	LS sepsis	44.5 (39.9–49.8)	59.5 (53.3–60.8)^†^	45.5 (40.7–62.1)
	HS sepsis	41.1 (37.3–51.8)	29.6 (25.9–44.7)	24.8 (23.1–35.5)
L_I+II_	LS sepsis	21.1 (19.7–24)	27.3 (26–30.3)^*†^	24.7 (21.4–29.2)
	HS sepsis	15.8 (14.6–21.7)	17.5 (14.5–20.1)	18 (15.3–20)
E_I+II_	LS sepsis	31.2 (29.6–35,3)	53.2 (41–56.4)^†^	46 (32.9–87.3)
	HS sepsis	38.9 (28.4–44.6)	35.2 (30.9–55.2)	31.2 (27.8–41.6)
E_II_	LS sepsis	31.2 (29.6–35.3)	53.2 (41–56.4)^*†^	45.9 (32.9–52.4)
	HS sepsis	38.9 (28.4–44.6)	24.9 (21.8–39.5)	21 (19.5–26.9)
C_IV_	LS sepsis	160.6 (120.2–192.9)^†^	175.8 (147.7–187.7)^†^	102.7 (90.6–152.4)
	HS sepsis	79.4 (69–103.4)	105 (91.7–116.1)	71.4 (56.2–78)
P_I_-L_I_	LS sepsis	31.6 (21.3–34.5)	34.8 (33–35.7)^†^	29 (25.3–38.1)
	HS sepsis	27.7 (26.8–35.7)	18.6 (17–23)^*^	13.2 (12.9–19.4)
P_I+II_-L_I+II_	LS sepsis	26.0 (19.4–27.7)	30.5 (25.5–34,3)^†^	25.0 (20.2–30.8)
	HS sepsis	27.4 (23.0–28.5)	12.7 (10.3–24.0)^*^	8.8 (7.8–16.4)
E_I+II_-L_I+II_	LS sepsis	32.2 (29.4–37.2)	46.3 (35.1–59.2)	42.8 (30.3–56)
	HS sepsis	31.8 (26.1–45.9)	19.7 (14.4–35.3)^*^	13.2 (12.5–21.6)
(P_I_-L_I_)/P_I_	LS sepsis	0.92 (0.90–0.93)	0.92 (0.86–0.96)^†^	0.85 (0.71–0.83)
	HS sepsis	0.95 (0.93–0.96)	0.90 (0.89–0.93)^*^	0.79 (0.78–0.79)
L_I+II_/P_I+II_	LS sepsis	0.46 (0.44–0.49)	0.50 (0.43–0.52)	0.50 (0.44–0.54)
	HS sepsis	0.38 (0.36–0.46)	0.56 (0.46–0.62)*	0.59 (0.53–0.66)
L_I+II_/E_I+II_	LS sepsis	0.41 (0.34–0.43)	0.39 (0.32–0.43)	0.37 (0.35–0.38)
	HS sepsis	0.32 (0.26–0.40)	0.42 (0.36–0.52)	0.52 (0.47–0.55)

All values shown are median with interquartile range. L_I_ —leak respiration (non-phosphorylating resting state) of complex I. P_I_—oxidative phosphorylation capacity (respiratory capacity of mitochondria in ADP saturated state) of complex I. P_I+II_—oxidative phosphorylation capacity (respiratory capacity of mitochondria in ADP saturated state) of complex I and II. L_I+II_—leak respiration (non-phosphorylating resting state) of complex I and II. E_I+II_—electron-transfer-pathway capacity (capacity when oxidation is not coupled to phosphorylation) of complex I and II. E_II_—electron-transfer-pathway capacity (capacity when oxidation is not coupled to phosphorylation) of complex II. C_IV_—complex IV activity. P_I_-L_I_—oxidative phosphorylation of complex I corrected for the leak respiration. P_I+II_-L_I+II_—oxidative phosphorylation of complex I and II corrected for the leak respiration. E_I+II_-L_I+II_—electron-transfer-pathway capacity of complex I and II corrected for the leak respiration. (P_I_-L_I_)/P_I_—control efficiency ratio of complex I (proportion of respiration directly coupled to ATP synthase by complex I). L_I+II_/P_I+II_—coupling control ratio of complex I and II representing uncoupling controlled by L and P. L_I+II_/E_I+II_—leak control ratio of complex I and II quantifying uncoupling controlled by L and E. LS—low severity. HS—high severity

*Significant change from the preceding time-point

^†^significant difference between groups

**Fig. 5:**
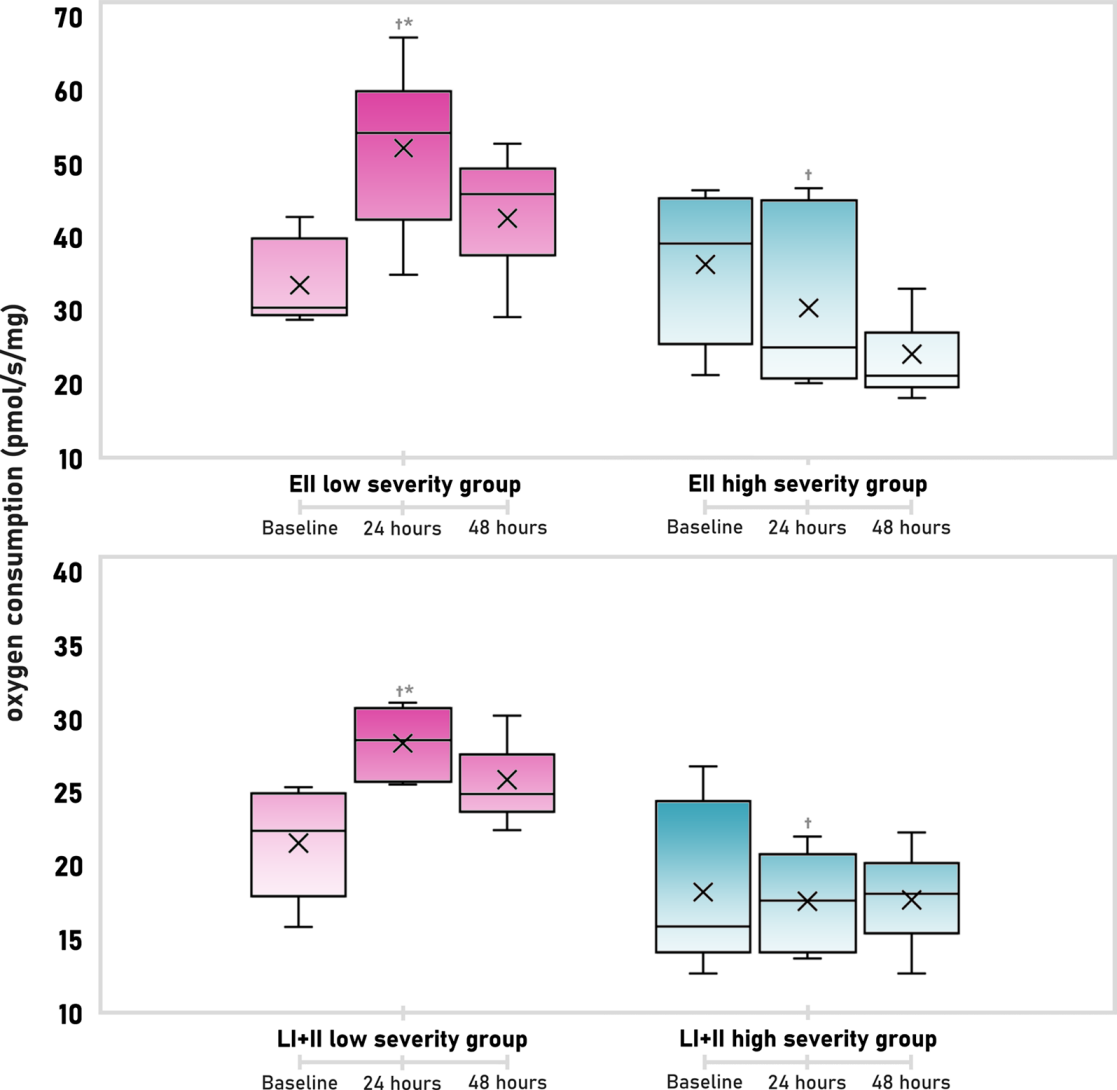
Selected respirometry variables in LS (low severity) and HS (high severity) sepsis group. EII—electron-transporting capacity of complex II; LI + II—LEAK respiration of complex I and II. * Significant change from the preceding time-point, ^†^ significant difference between groups

In the first 24 h of low severity sepsis oxygen consumption in the leak state and maximum electron system transfer capacity increased suggesting increased mitochondrial activity with higher oxygen demands for compensation of the proton leak, proton slip and cation cycling. No other parameter reached statistical significance, OXPHOS and ET capacities for complexes I and II were border-line non-significant, although they both were increased (Fig. 5)*.*

**Fig. 6:**
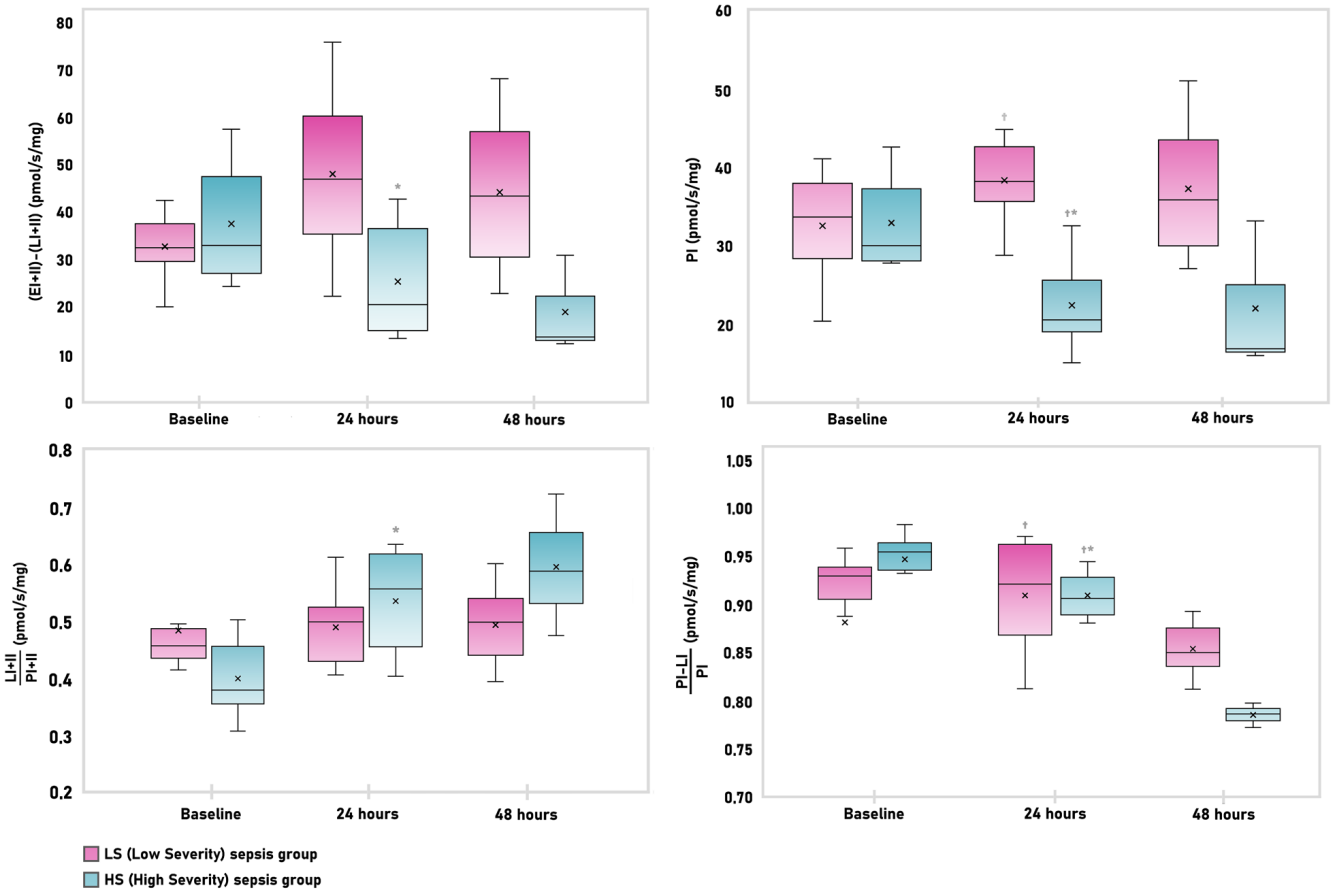
Selected respirometry variables in in LS (low severity) and HS (high severity) sepsis group. (EI + II)-(LI + II)—E-L net ET capacity (the capacity of respiratory system potentially available for ATP synthesis); (PI-LI)/PI—P-L control efficiency (the proportion of respiration directly coupled to ATP synthase); PI—OXPHOS capacity of complex I; (LI + II)/(PI + II)—coupling control ration (sensitive marker of uncoupling). * Significant change from the preceding time-point, ^†^ significant difference between groups

High severity sepsis was associated with a significant decrease in OXPHOS capacity for complex I and in absolute and relative number of protons used in ATP synthesis by complexes I and II. The capacity of the respiratory system (E-L net ET capacity) and the proportion of respiration coupled to ATP synthesis was decreased (control efficiency ratio). Mitochondrial respiration uncoupling was increased (leak control ratio). Although the changes observed in the subsequent 24 h did not reach statistical significance, a trend towards further deterioration could be noted in parameters reflecting higher degree of uncoupling. Comparing high severity and low severity sepsis, mitochondrial respiratory function parameters were significantly lower in the HS group, indicating decrease in both transport and phosphorylating functions. Absolute and relative amount of oxygen utilized in phosphorylation of ADP to ATP by ATP synthase was decreased with higher uncoupling (Fig. 6).

### Renal high-resolution respirometry in the end-point kidney specimen

Results of high-resolution respirometry in the end-point kidney specimen are summarized in Table 3 and the same results adjusted to the activity of citrate synthase (Krebs cycle enzyme) in Table A4 (see *Additional material*).

**Table 3:** End-point kidney specimen high-resolution respirometry results

Respirometry parameters (pmol O_2_/s/mg)	Group	Renal cortex	Renal medulla
L_I_	LS sepsis	10.8 (7.2–11.3)^*^	3.6 (2.6–4.5)^*^
	HS sepsis	9.1 (6.0–11.3)^*^	3.6 (2.4–5.1)^*^
P_I_	LS sepsis	28.9 (28.1–48.5)^*^	12.9 (11.7–14.5)^*^
	HS sepsis	28.7 (19.5–33.0)^*^	12.8 (6.7–16.4)^*^
P_I+II_	LS sepsis	57.4 (43.1–68.7)^*^	18.3 (15.8–22.0)^*^
	HS sepsis	42.7 (41.2–44.5)^*^	18.1 (14.1–20.9)^*^
L_I+II_	LS sepsis	26.0 (22.3–33.2)^*^	10.3 (9.1–11.6)^*^
	HS sepsis	32.3 (28.7–34.2)^*^	11.8 (8.6–14.9)^*^
E_I+II_	LS sepsis	54.3 (48.2–79.1)^*^	23.2 (19.6–25.8)^*^
	HS sepsis	48.7 (47.4–55.4)^*^	20.1 (17.1–24.8)^*^
E_II_	LS sepsis	44.4 (39.4–61.4)^*^	15.9 (13.2–17.7)^*^
	HS sepsis	41.7 (37.6–46.5)^*^	16.5 (13.4–19.6)^*^
C_IV_	LS sepsis	113.0 (102.2–126.6)^*^	69.8 (62.4–82.6)^*^
	HS sepsis	115.7 (112.5–120.8)^*^	71.7 (60.5–85.3)^*^
P_I_-L_I_	LS sepsis	22.4 (18.5–36.0)^*^	9.0 (7.9–11.3)^*^
	HS sepsis	19.9 (13.5–23.8)^*^	7.8 (3.7–11.7)^*^
P_I+II_-L_I+II_	LS sepsis	25.7 (21.2–38.3)^*†^	8.1 (7.1–10.0)^*^
	HS sepsis	10.4 (7.0–15.6)	5.6 (5.3–6.9)
E_I+II_-L_I+II_	LS sepsis	29.4 (24.1–49.0)^*^	12.5 (10.0–14.9)^*^
	HS sepsis	18.5 (13.3–25.9)^*^	8.3 (7.2–8.7)^*^
(P_I_-L_I_)/P_I_	LS sepsis	0.8 (0.7–0.8)	0.7 (0.6–0.8)
	HS sepsis	0.7 (0.6–0.7)	0.6 (0.5–0.7)
L_I+II_/P_I+II_	LS sepsis	0.5 (0.4–0.5)	0.6 (0.5–0.6)
	HS sepsis	0.6 (0.5–0.8)	0.6 (0.6–0.6)
L_I+II_/E_I+II_	LS sepsis	0.4 (0.4–0.5)	0.4 (0.4–0.5)
	HS sepsis	0.6 (0.5–0.7)	0.6 (0.5–0.6)

All values shown are median with interquartile range. Individual variables described in Table 6. LS—low severity HS—high severity

*Significant difference between cortex and medulla

†significant difference between groups

**Table 4:** Citrate synthase activity in renal cortex and medulla

Citrate synthase activity (IU/g)	Group	Renal cortex	Renal medulla
	LS sepsis	12.7 (11.3–13.9)^* †^	3.8 (3.4–5.0)^*^
	HS sepsis	8.4 (5.5–9.9)^†^	7.1 (3.1–9.0)

All values shown are median with interquartile range

LS—low severity. HS—high severity

*Significant difference between cortex and medulla

†significant difference between groups

Compared to cortex, renal medulla had significantly lower oxidative phosphorylation capacity and electron-transport system activity in both low and high severity sepsis groups. The proportion of respiration directly coupled to ATP synthesis (P-L net OXPHOS capacity) was higher in the cortex of LS sepsis. Citrate synthase (CS) activity in renal medulla was significantly lower compared to the cortex in the low severity septic animals. In the cortex, CS activity was significantly lower in HS compared to LS group, whereas in the medulla no difference was noted between LS and HS animals. When adjusted to the CS activity, there was a trend towards a lower mitochondrial activity in medulla of HS septic animals compared to LS sepsis, although this did not reach statistical significance (*Additional material, *Table A5).

**Table 5:** Western blot analysis of mitogenesis and mitophagic flux markers

Protein	LS sepsis	HS sepsis
PGC-1-alfa	1.25 (1.21–1.38)^†^	1.06 (0.89–1.12)
LC3 A/B I	1.68 (1.57–2.22)	2.13 (1.85–3.01)
LC3 A/B II	0.77 (0.7–0.86)	1.28 (0.98–1.33)

All values shown are median with interquartile range. Results normalized to total protein. PGC-1-alfa—peroxisome proliferator activated receptor-gamma-co-activator 1-alfa. LC3 A/B—microtubule-associated proteins light chain 3 A/B. LS—low severity. HS—high severity

^†^Significant difference between groups

### Western blotting of proteins involved in mitochondrial biogenesis and degradation

The results of the Western blot analysis of PGC-1-alfa and LC3 A/B are shown in Table 5*.*

**Fig. 7:**
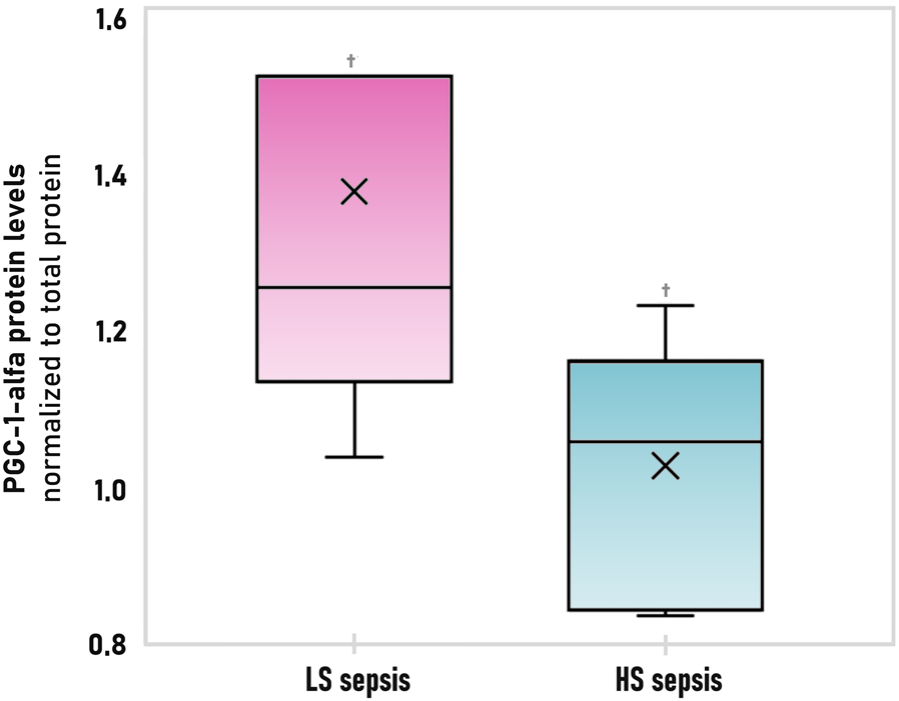
PGC-1-alfa expression in dynamic model of LS (low severity) sepsis in pink and HS (high severity) sepsis in blue. PGC-1-alfa—peroxisome proliferator activated receptor-gamma-co-activator 1-alfa. ^†^ Significant difference between groups

PGC-1-alfa, a key regulator of energy metabolism, was significantly lower in the HS sepsis group. Levels of both LC3 A/BI and LC3 A/BII that might indicate changes in mitophagic flux were not different comparing both groups (Fig. 7).

## Discussion

In this randomized controlled study, we gathered unique sequential data from large septic animals with different severity of inflammation and paces of sepsis progression. Importantly, we coupled those results with traditional parameters of renal physiology to avoid misinterpretation of complex mitochondrial data. Compared to the low severity sepsis group, we observed an early and significant decrease in oxidative phosphorylation capacity together with an increased uncoupling in high severity illness during the first 24 h. These changes preceded alterations in renal haemodynamics, since renal blood flow significantly fell only afterwards. Moreover, serum creatinine increased significantly during the first day, suggesting that renal injury was not solely related to haemodynamic changes. A significant decrease in oxidative phosphorylation and citrate synthase activity was observed in the renal medulla, possibly indicating a difference in mitochondrial response along the nephron. PGC-1-alfa, a marker of mitochondrial biogenesis, was significantly decreased in the HS sepsis group. Several authors have suggested a potential role for mitochondrial dysfunction in sepsis-driven multiorgan failure [22, 23]. However, it is still unclear whether the organ dysfunction is a result of mitochondria damage or whether mitochondria are just victims of (sub)cellular response to inflammation. This “cell-adaptive organ-maladaptive” strategy [15] might explain a rapid recovery of renal functions in some patients with septic acute kidney injury after resolution of systemic infection and a lack of cell death in renal biopsies of patients with sepsis and acute renal failure. Precise pathophysiology of the interaction between mitochondrial bioenergetics and inflammation remains incompletely understood. Krawczyk et al. showed higher glycolysis activity accompanied by hyperlactatemia not associated with hypoxia after stimulation of TLRs of innate immunity in an animal model [24]. Other authors then pointed out similar metabolic reprogramming in activated NK cells, proinflammatory macrophages and T and B-cells, [25] and the relationship between inflammation and metabolism appears to be bidirectional [26–29]. Therefore, it appears that at least in some phenotypes of sepsis, cell metabolism is altered early in the course of infection, causing a suppression of energy-consuming processes other than those that are essential for cell survival. The subcellular background for this is probably instrumented by the complex immune-metabolic crosstalk between two main pathways integrating input from various upstream processes—AMPK pathway and Akt/mTOR/HIF-1-alfa pathway. AMPK is an enzyme regulated primarily by the cellular energy status, but also via cGMP, which serves as a main product of NO synthase activation in inflammation [30–32]. AMPK mobilizes energetic substrates and restrains synthetic energy-consuming processes [33]. To maximize mitochondrial efficacy, AMPK stimulates mitogenesis by PGC-1-alfa activation [34]. However, during systemic inflammation, PGC-1-alfa is downregulated by proinflammatory cytokines, as well as PAMP themselves [35]. To counterbalance AMPK actions, mTOR senses cellular nutrients, energy and oxygen levels and acts as a switch to the anabolic cellular milieu [36]. Glycolytic flux is also increased, but the mitochondrial respiration is mitigated [37–39]. Inter-mitochondrial communication and homeostasis is also influenced by mTOR and AMPK. AMPK down-regulates mTOR and thus potentiates mitophagy [40]. Formation of mitophagosome is mediated by microtubule-associated proteins 1A/1B light chain 3B, referred to as LC3 proteins, which may serve as a marker of mitophagic flux [41]. In systemic inflammation yet another pathway, HIF-1-alfa, become activated and upregulates anaerobic glycolysis, vascular neogenesis and induces mitophagy [42–45].

Our data support the thesis of a close connection between mitochondrial (dys)function and sepsis. Renal tubular cells expressing PRRs [46] probably undergo similar immuno-metabolic changes as immune cells and enter self-preserving state limiting tubular functions, including creatinine secretion. Furthermore renin–angiotensin system is disrupted, since the highest concentrations of angiotensin-converting enzyme are localized in the proximal tubular brush border, which is typically dysfunctional during sepsis [47, 48]. However, it is obvious that renal mitochondrial response profoundly differs depending on severity of inflammatory insult. This is reflected in PGC-1-alfa down-regulation and dampened citrate synthase activity as well as mitochondrial respirometry parameters in high-severity septic animals. We did not observe a significant difference in LC3 proteins, correlating with suspected preferential mTOR stimulation. Those data need to be interpreted in the context of several other studies showing conflicting results regarding mitochondrial functions [49, 50]. A similar study investigating renal cortical mitochondria in relation to oxygen consumption in an ovine model of sepsis was recently published by Luther et al. [51]. A decrease in the relationship between renal VO_2_ and renal sodium transport was observed in septic AKI, yet neither mitochondrial uncoupling nor a decrease in the efficiency of mitochondrial electron-transport chain was demonstrated, which contradicts our results. However, the experimental protocol deviated significantly from the one utilized in our study. Specifically, the infusion of bacteria was titrated against the physiological response by the investigators at 6- and 12-h intervals. While this may result in a seemingly homogeneous population of septic animals based on their phenotype (i.e. haemodynamic response), the endotype of sepsis and mitochondrial renal functions will be profoundly different. Consequently, it can be hypothesized that the mitochondrial response varies depending on the severity of the inflammatory insult. Heterogeneity of septic AKI endotypes might be the main reason for inconsistent findings mentioned above. To put it another way, not all patients with sepsis develop acute kidney injury based on metabolic reprogramming and mitochondrial dysfunction. Renal dysfunction in sepsis is mediated by multiple mechanisms and endotype of septic AKI driven mainly by cell reprogramming likely manifest as a specific phenotype of AKI with a potentially prompt recovery of renal functions. These initially adaptational changes progress to definitive organ dysfunction via different mechanisms, but oxidative stress associated with disrupted renal mitochondria oxygen handling seems to play a significant role [52].

Our study had several limitations. Propofol, used as a sedative, is known to have a mitochondria-interfering effect [53]. The precise mechanism responsible for propofol-related mitochondrial toxicity remains elusive. In animal models, propofol uncouples oxidative phosphorylation and inhibits electron-transport chain [54, 55]. Furthermore, propofol likely inhibits the activity of carnitine palmitoyl transferase, an essential mitochondrial enzyme in the beta-oxidation of long-chain fatty acids [56]. The subsequent accumulation of free-fatty acids is considered to be proarrhythmogenic [57]. Although this has traditionally been regarded as an issue of prolonged and high doses (clinically known as PRIS—propofol-related infusion syndrome), there is some evidence suggesting a mitochondrial disruptive effect several hours after the initiation of an infusion even at clinically relevant tissue concentrations [58]. Therefore, we cannot exclude a possible influence of propofol in our analysis. However, there was no difference in propofol or fentanyl doses between the low and high severity groups and no adjustments were made in order to mitigate haemodynamic effect of analgosedation during the experiment. Therefore, we assume that any potential influence is the same in both groups. The definition of AKI used in our study is mainly based on serum creatinine levels, which we believe to be a reliable marker in the porcine sepsis model [59]. Although some authors consider plasma creatinine a poor marker of glomerular filtration rate in pigs [60], we observed a significant time-dependent changes and between-group differences, making our observations relevant even when considering potential limitations of this biomarker of renal injury. Interestingly, we did not observe oliguria in our animals, although typically septic AKI is associated with low urine output. We do not have a clear explanation for this finding, and we can only speculate to what extent altered tubular re-absorption capacity may have contributed to the increased diuresis. Kidney tissue biopsies represent heterogeneous group of different cell types in different immuno-metabolic states and the proportion of those may differ among samples. Renal tissue injury is not uniform throughout the kidney during AKI, zones of inflammatory, hypoxic nephrons might coexist with clusters of relatively preserved nephrons [61]. Furthermore, our sample size was relatively small mainly due to premature death occurring in the sepsis group, introducing possible bias tackling missing data. We did not have a healthy control group. However, our research group had recently published a study with an experimental protocol comparable to HS-sepsis group in the current study and this study, which was focused on myocardial function in porcine septic shock, included a sham operated control group. Although the results cannot be directly extrapolated to the kidneys, the mitochondrial respiration was suppressed in septic hearts compared to control animals, supporting our findings in the current study [62]. Finally, we studied young, otherwise healthy animals. Several risk factors like aging, hypertension, diabetes and chronic kidney disease are known to alter mitochondrial functions and increase the susceptibility to AKI in sepsis. Understanding the differences in renal mitochondrial response in the presence of pre-existing comorbidities in comparison to healthy kidneys would be informative.

## Conclusions

Mechanisms behind septic acute kidney injury remain largely elusive. Our study indicates that renal mitochondrial response profoundly differs depending on the severity of infectious insult and support the thesis of initially reversible renal cell metabolism reprioritization being responsible for some endotypes of early septic acute kidney injury. The framework of septic AKI being a group of heterogenous entities rather than a single disease advocates for a paradigm shift in the upcoming studies. Complex, dynamic and clinically relevant large animal trials evaluating specific subgroups of septic acute kidney injury, integrating sequential tissue molecular analyses, complex physiological monitoring and human-like intensive care are necessary.

## Supplementary Information

Additional file 1: Table A1. Characterization of sepsis model—systemic hemodynamics. All values shown are median with interquartile range. Table A2. Inflammatory markers and key metabolic intermediate ratios data. All values shown are median with interquartile range. Table A3. Renal hemodynamics. All values shown are median with interquartile range. Table A4. End-point kidney specimen high-resolution respirometry results adjusted to the activity of citrate synthase.

## Data Availability

The datasets supporting the conclusions of this article are available from the corresponding author upon request.

## Abbreviations

AKI: Acute kidney injury
AMPK: AMP-activated protein kinase
ATP/ADP: Adenosine triphosphate/adenosine diphosphate
cGMP: Cyclic guanosine monophosphate
CIV: Complex IV activity
CO: Cardiac output
CVP: Central venous pressure
DO: Oxygen delivery
E (ETS capacity): Electron transport system capacity
HIF-1-alfa: Hypoxia inducible factor-alfa
HR: Heart rate
HS sepsis: High severity sepsis
IL-6: Interleukin 6
L (LEAK respiration): Leak state respiration
LC3 Proteins: Microtubule-associated proteins light chain 3
LS Sepsis: Low severity sepsis
MAP: Mean arterial pressure
MPAP: Mean pulmonary artery pressure
mTOR: Mammalian target of rapamycin
NK cell: Natural killer cell
NO: Nitric oxide
O2ER: Oxygen extraction ratio
P (OXPHOS capacity): Oxidative phosphorylation capacity
PAMP: Pathogen associated molecular pattern
PAWP: Pulmonary artery wedge pressure
PEEP: Positive end expiratory pressure
PGC-1-alfa: Peroxisome proliferator activated receptor-gamma-co-activator 1-alfa
PiCCO: Pulse contour cardiac output
PRR: Pattern recognition receptor
PVDF: Polyvinylidene difluoride
Qren: Renal blood flow
RVP: Renal venous pressure
RVR: Renal vascular resistance
SOFA: Sequential Organ Failure Assessment
SVR: Systemic vascular resistance
SVR: Systemic vascular resistance
TBST: Tris-buffered saline 0,1% Tween
TLR: Toll like receptor
TNF-alfa: Tumour necrosis factor-alfa
TP: Time point
VO2: Oxygen consumption
