# Supplementary material for: Renal mitochondria response to sepsis: a sequential biopsy evaluation of experimental porcine model
Source: Intensive Care Med Exp. 2025 Feb 22;13:25. doi: 10.1186/s40635-025-00732-0 (PMC11846788; doi:10.1186/s40635-025-00732-0)
Supplement: Supplementary file 1 — Additional file 1: Table A1. Characterization of sepsis model—systemic hemodynamics. All values shown are median with interquartile range. Table A2. Inflammatory markers and key metabolic intermediate ratios data. All values shown are median with interquartile range. Table A3. Renal hemodynamics. All values shown are median with interquartile range. Table A4. End-point kidney specimen high-resolution respirometry results adjusted to the activity of citrate synthase. [file 40635_2025_732_MOESM1_ESM.docx]

**Supplement**

**Additional material 1 – Measurements, monitoring, and sampling details**

Pigs were monitored by Mindray BeneVision T9. Systemic hemodynamic parameters included direct measurement of ECG heart rate (HR), mean arterial pressure (MAP), central venous pressure (CVP), mean pulmonary artery pressure (MPAP) and pulmonary artery wedge pressure (PAWP). Cardiac output (CO) was measured by the thermodilution Stewart-Hamilton principle. Based on systemic parameters systemic vascular resistance (SVR) was calculated as:

$$SVR (dynes\times sec\times{cm}^{-5})=80\times\frac{MAP \left( mmHg \right)-CVP(mmHg)}{CO (l/min)}$$

Delivery of oxygen (DO_2_) and oxygen consumption (VO_2_) were calculated as:

$${DO}_{2}(ml/min)=CO(l/min)\times\left[ Hb (g/l)\times1.34\times\frac{S_{a}O_{2}}{100}+p_{a}O_{2}(kPa)\times0.225 \right]$$

$${VO}_{2}(ml/min)=CO (l/min)\times\left\{ Hb (g/l)\times1.34\times\frac{S_{a}O_{2}-S_{v}O_{2}}{100}+\left[ p_{a}O_{2}\left( kPa \right)-p_{v}O_{2}\left( kPa \right) \right]\times0.225 \right\}$$

Oxygen extraction ratio (O_2_ER) was then derived as:

$$O_{2}ER=\frac{{VO}_{2} (ml/min)}{{DO}_{2} (ml/min)}$$

PiCCO derived parameters – intrathoracic blood volume (ITBV), stroke volume (SV), left ventricle stroke work (LVSW) and right ventricle stroke work (RVSW) were recorded.

Regional hemodynamic parameters consisted of renal artery blood flow (Qren) obtained by ultrasound probe (Transonic Systems) and renal venous pressure (RVP). Renal vascular resistance (RVR) was calculated as:

$$RVR (mmHg/l/min/kg)=\frac{MAP \left( mmHg \right)-RVP(mmHg)}{Qren (l/min/kg)}$$

Complete blood count, serum creatinine, urea, bilirubin aspartate aminotransferase (AST), alanine aminotransferase (ALT), alkaline phosphatase (ALP), total protein and serum glucose levels were obtained in each time-point. Bed-side measurement (ABL90 Flex Plus, Radiometer) included arterial and mixed venous blood samples analysed for pH, pO_2_, pCO_2_, haemoglobin saturation, base excess and lactate.

**Additional material 2 – Evaluation of mitochondrial respiration**

Mitochondrial respiration was assessed using high-resolution respirometry (oxygraph O2k, Oroboros, Innsbruck, Austria). Whole tissue samples of swine kidney cortex were mechanically permeabilized and washed in mitochondrial respiration medium MIR05 composed of 0.5 mmol/L MgCl_2_·6H_2_O, 60 mmol/L potassium lactobionate, 20 mmol/L taurine, 10 mmol/L KH_2_PO_4_, 20 mmol/L HEPES, 110 mmol/L sucrose, and 1 g/L fatty acid free bovine serum albumin, pH 7.0 (19). Samples were placed into pre-calibrated oxygraph chambers containing MiR05 solution with oxygen content equilibrated with air at 37 °C. After closing the chambers, oxygen consumptions were analysed on-line by DatLab software (Oroboros Instruments, Innsbruck, Austria) as negative time derivative of oxygen concentration in the chamber and adjusted to the ROX (residual oxygen consumption) state – oxygen consumption after complete inhibition of the electron transfer system. To evaluate functional state of mitochondrial respiration a protocol with sequential titration of substrates and inhibitors of electron-transferring complexes was performed. LEAK respiration (L; non-phosphorylating resting state) was induced by addition of substrates providing electrons to complex I – glutamate (10 mmol/L), malate (2 mmol/L), and pyruvate (5 mmol/L). OXPHOS I capacity (PI) as a surrogate of the electron system transfer through complexes I, III and IV chemiosmotically coupled to the phosphorylation of ADP by the ATP synthase was measured after 5 mmol/L ADP and 10 µmol/L cytochrome c. OXPHOS I+II capacity (PI+II) was determined after 10 mmol/L succinate, ETS I+II capacity (EI+II; uncoupled respiration estimating the electron transport system capacity when oxidation is not coupled to phosphorylation) after titrations of trifluorocarbonylcyanide phenylhydrazone (FCCP; 0.05 µmol/L titration steps), EII after addition of a complex I inhibitor 0.5 μmol/L rotenone. Complex III inhibition by 2.5 μmol/L antimycin A initiated the state ROX and simultaneous injection of 0.5 mmol/L N,N,N′,N′-tetramethyl-p-phenylenediamine dihydrochloride, TMPD, and 2 mmol/L ascorbate enabled estimation of complex IV activity (CIV). In our study, each value was measured in duplicates at least; the results are mean of those measurements. Several calculated parameters and ratios were determined. P-L (P-L net OXPHOS capacity) reflects the amount of oxygen utilized in phosphorylation of ADP to ATP by ATP-synthase; E-L (E-L net ET capacity) indicates the capacity of respiratory system potentially available for ATP synthesis. P-L/P (P-L control efficiency) is the ratio of oxygen consumed for ATP production (P-L net OXPHOS capacity) to total OXPHOS capacity, showing the proportion of respiration directly coupled to ATP synthase. E-L/E (E-L coupling efficiency) predicts the potential capacity of ADP phosphorylation. L/P (coupling-control ratio) is a ratio of LEAK respiration to OXPHOS capacity and represents a rough marker of uncoupling controlled by L and P. L/E (leak-control ratio) is LEAK state over ET capacity and might serve as an index quantifying the uncoupling process (controlled by L and E). A kidney specimen taken at the end of the experiment was analysed for total oxygen consumption. Cortex and medulla were examined separately for (a) oxygen consumption per milligram of the whole tissue sample and (b) oxygen consumption per activity of citrate-synthase, one of enzymes of the tricarboxylic acid cycle. The end-point kidney samples were frozen and then the enzymatic activity of citrate synthase was measured simultaneously in all samples at the end of the study – 100 µL of sample was mixed with 900 µL of the assay medium composed of 0.1 mmol/L 5,5-dithio-bis- (2-nitrobenzoic) acid, 0.25% triton-X, 0.5 mmol/L oxaloacetate, 0.31 mmol/L acetyl coenzyme A, 5 µmol/L EDTA, 5 mmol/L triethanolamine hydrochloride, and 0.1 mol/L tris-HCl, pH 8.1. The rate of absorbance change was determined by spectrophotometry at 412 nm and 30 °C over 20 s. Enzyme activity was expressed in IU/g tissue wet weight (20). If not stated otherwise, all chemicals were purchased from Sigma-Aldrich (Prague, Czech Republic).

**Additional material 3 – Systemic hemodynamics and inflammatory parameters**

|  | **Group** | **Baseline** | **24 hours** | **48 hours** |
| --- | --- | --- | --- | --- |
| CVP (mmHg) | LS sepsis | 10 (9–10) | 11 (11–14) * | 15 (13–18) *^∆^ |
|  | HS sepsis | 10 (8–11) | 14 (13–15) * | 12 (11–14) |
| MPAP (mmHg) | LS sepsis | 20 (20–23) | 27 (26–30) * | 30 (29–36) ^∆^ |
|  | HS sepsis | 21 (20–24) | 30 (26–32) * | 31 (29–36) |
| PAWP (mmHg) | LS sepsis | 9 (9–11) | 11 (11–12) | 14 (13–17) * ^∆^ |
|  | HS sepsis | 11 (9–11) | 12 (11–14) | 14 (13–15) |
| CO (mL/kg/min) | LS sepsis | 68 (61–75) | 90 (87–124) * ^†^ | 100 (72–110) |
|  | HS sepsis | 88 (75–91) | 162 (136–173) * ^†^ | 105 (94–123) ^∆^ |
| SVR (dyne∙s/cm^5^) | LS sepsis | 2121 (1930–2380) | 1185 (1154–1389) * | 1364 (1102–1578) ^∆^ |
|  | HS sepsis | 1738 (1410–2143) | 837 (709–1166) * | 582 (575–780) |
| DO2-S (mL/min/kg) | LS sepsis | 7 (7–10) | 9 (9–12) ^†^ | 11 (9–11) ^†^ |
|  | HS sepsis | 8 (8–10) | 19 (14–21) * ^†^ | 23 (18–24) ^†^ |
| VO2-S (mL/min/kg) | LS sepsis | 3.8 (3.5–3.9) | 4.4 (4.3–4.7) ^†^ | 4 (3.9–4.5) |
|  | HS sepsis | 4.3 (4.0–4.6) | 6.3 (5.6–8.1) * ^†^ | 5.8 (5.4–6.7) |
| O2EXTR-S (%) | LS sepsis | 54 (43–55) | 44 (37–47) | 43 (41–47) |
|  | HS sepsis | 49 (40–55) | 41 (35–47) | 23 (23–38) |

**Table A1:** Characterization of sepsis model – systemic hemodynamics. All values shown are median with interquartile range. MAP – Mean Arterial Pressure; CVP – Central Venous Pressure; MPAP – Mean Pulmonary Artery Pressure; PAWP – Pulmonary Artery Wedge Pressure; CO – Cardiac Output; SVR – Systemic Vascular resistance; DO2-S – systemic oxygen delivery; VO2-S – systemic oxygen consumption; O2EXTR-S – systemic oxygen extraction ratio. LS – Low Severity. HS – High Severity. * Significant change from the preceding time-point ^∆^Significant difference from the baseline ^†^ Significant difference between groups

|  | **Group** | **Baseline** | **24 hours** | **48 hours** |
| --- | --- | --- | --- | --- |
| IL-6  (pg/g of protein) | LS sepsis | 76 (51–80) | 469 (324–838) * ^†^ | 195 (113–233) * ^∆^ |
|  | HS sepsis | 95 (67–106) | over 2000* ^†^ | over 2000 (1079–over 2000) ^∆^ |
| TNF-alpha (pg/g of protein) | LS sepsis | 72 (51–80) | 156 (123,6–227) * ^†^ | 113 (73–127) * ^∆†^ |
|  | HS sepsis | 83 (67–133) | 292 (201–447) * ^†^ | 268 (197–588) ^†^ |
| lactate-V (mmol/L) | LS sepsis | 1.1 (1.0–1.2) | 0.8 (0.7–1.4) | 1.0 (0.8–1.1) |
|  | HS sepsis | 1.0 (0.9–1.2) | 1.6 (1.2–4.1) | 2.1 (1.1–2.8) |
| pyruvate-V (mmol/L) | LS sepsis | 0.06 (0.04–0.16) | 0.07 (0.03–0.08) | 0.05 (0.02–0.07) |
|  | HS sepsis | 0.14 (0.11–0.17) | 0.09 (0.05–0.18) | 0.10 (0.06–0.17) |
| lactate/pyruvate ratio | LS sepsis | 17 (8–24) | 16 (10–25) | 15 (11–22) |
|  | HS sepsis | 9 (5–14) | 22 (13–26) | 14 (8–18) |

**Table A2:** Inflammatory markers and key metabolic intermediate ratios data. All values shown are median with interquartile range. Values of IL-6 over 2000 pg/g are unmeasurable with our analysis kit, statistics conducted with the maximal measurable value. V – renal vein sampling site. LS – Low Severity. HS – High Severity. * Significant change from the preceding time-point ^∆^Significant difference from the baseline ^†^ Significant difference between groups

**Additional material 4 – Renal hemodynamics and other parameters**

|  | **Group** | **Baseline** | **24 hours** | **48 hours** |
| --- | --- | --- | --- | --- |
| Qren  (mL/min/kg) | LS sepsis | 5 (4–7) | 6 (5.2–6,5) | 6.9 (5.5–9.1) ^†^ |
|  | HS sepsis | 4.7 (4.1–5) | 3.4 (2,2–4,8) | 0.7 (0.5–0.8) * ^∆†^ |
| RVP (mmHg) | LS sepsis | 12 (10.5–12.5) | 15 (13–15.5) | 18 (14.5–19) ^*∆^ |
|  | HS sepsis | 11.5 (10.3–13.5) | 16 (14.3–18.5) ^*^ | 14 (14–15.5) |
| delta RVR (%) | LS sepsis | 0 | 74 (67–80) | 66 (51–76) ^*∆†^ |
|  | HS sepsis | 0 | 9 (8–19) | 49 (34–59) ^*†^ |
| DO2-R (mL/min/kg) | LS sepsis | 0.6 (0.5–0.9) | 0.6 (0.5–0.8) | 0.7 (0.6–0.8) ^†^ |
|  | HS sepsis | 0.5 (0.4–0.6) | 0.5 (0.3–0.6) | 0.1 (0.1–0.3) ^†^ |
| VO2-R (mL/min/kg) | LS sepsis | 0.2 (0.1–0.2) | 0.2 (0.1–0.2) | 0.2 (0.1–0.2) |
|  | HS sepsis | 0.2 (0.1–0.2) | 0.2 (0.1–0.2) | 0 (0–0.1) |
| O2EXTR-R (%) | LS sepsis | 26 (20–32) | 30 (23–34) | 29 (20–35) |
|  | HS sepsis | 33 (20–42) | 28 (25–36) | 28 (19–33) |

**Table A3:** Renal hemodynamics. All values shown are median with interquartile range. Qren – Renal Blood Flow; RVP – Renal Venous Pressure; RVR – Renal Venous Resistance; DO2-R – renal oxygen delivery; VO2-R – renal oxygen consumption; O2EXTR-R – renal oxygen extraction ratio; LS – Low Severity. HS – High Severity. * Significant change from the preceding time-point ^∆^Significant difference from the baseline ^†^ Significant difference between groups

**Additional material 5 – Results of high-resolution respirometry in the end-point kidney specimen adjusted to the activity of citrate synthase**

| **Respirometry Parameters (pmol O_2_/s/mIU)** | **Group** | **Renal cortex** | **Renal medulla** |
| --- | --- | --- | --- |
| L_I_ | LS sepsis | 0.8 (0.7–0.9) | 0.7 (0.5–1.0) |
|  | HS sepsis | 1.2 (0.8–1.9) | 0.6 (0.5–1.3) |
| P_I_ | LS sepsis | 2.8 (2.2–3.6) | 3.2 (2.8–3.6) |
|  | HS sepsis | 3.5 (3.1–4.3) | 1.9 (1.7–2.0) |
| P_I+II_ | LS sepsis | 4.7 (4.0–5.1) | 4.7 (4.1–4.8) |
|  | HS sepsis | 5.6 (4.6–8.5) | 2.6 (2.4–5.8) |
| L_I+II_ | LS sepsis | 2.2 (1.9–2.5) ^†^ | 2.4 (2.1–2.7) |
|  | HS sepsis | 3.6 (3.0–5.8) ^†^ | 1.7 (1.6–3.4) |
| E_I+II_ | LS sepsis | 5.1 (4.2–5.9) | 5.5 (4.6–6.1) |
|  | HS sepsis | 6.8 (5.8–8.8) | 3.0 (2.8–5.9) |
| E_II_ | LS sepsis | 3.8 (3.4–4.8) | 3.8 (3.4–4.0) |
|  | HS sepsis | 5.0 (4.7–6.7) | 2.4 (2.1–3.8) |
| C_IV_ | LS sepsis | 9.2 (8.3–11.0) ^* †^ | 15.9 (15.3–19.9) ^*^ |
|  | HS sepsis | 12.3 (12.1–21.6) ^†^ | 10.7 (9.8–28.3) |
| P_I_-L_I_ | LS sepsis | 2.2 (1.5–2.7) | 2.3 (1.8–2.7) |
|  | HS sepsis | 2.4 (1.4–3.2) ^*^ | 0.9 (0.9–2.1) ^*^ |
| P_I+II_-L_I+II_ | LS sepsis | 2.3 (1.9–2.9) | 1.8 (1.6–2.6) |
|  | HS sepsis | 1.4 (0.7–2.6) | 0.9 (0.9–2.1) |
| E_I+II_-L_I+II_ | LS sepsis | 2.9 (2.0–3.6) | 2.6 (2.3–3.8) |
|  | HS sepsis | 2.6 (1.7–3.3) | 1.2 (0.6–1.6) |
| (P_I_-L_I_)/P_I_ | LS sepsis | 0.06 (0.05–0.06) ^*^ | 0.16 (0.15–0.19) ^*^ |
|  | HS sepsis | 0.09 (0.07–0.10) | 0.10 (0.08–0.21) |
| L_I+II_/P_I+II_ | LS sepsis | 0.04 (0.03–0.04) ^*^ | 0.13 (0.11–0.16) ^*^ |
|  | HS sepsis | 0.08 (0.05–0.13) | 0.09 (0.08–0.24) |
| L_I+II_/E_I+II_ | LS sepsis | 0.04 (0.03–0.04) ^*†^ | 0.12 (0.09–0.13) ^*^ |
|  | HS sepsis | 0.07 (0.06–0.13) ^†^ | 0.08 (0.07–0.21) |

**Table A4:** End-point kidney specimen high-resolution respirometry results adjusted to the activity of citrate synthase. All values shown are median with interquartile range. LS – Low Severity. HS – High Severity. *Significant difference between cortex and medulla; †Significant difference between groups
